# Complex spatial clonal structure in the macroalgae *Fucus radicans* with both sexual and asexual recruitment

**DOI:** 10.1002/ece3.1629

**Published:** 2015-09-09

**Authors:** Angelica Ardehed, Daniel Johansson, Ellen Schagerström, Lena Kautsky, Kerstin Johannesson, Ricardo T. Pereyra

**Affiliations:** ^1^ Department of Biology and Environmental SciencesUniversity of GothenburgBox 463, SE 405 30GothenburgSweden; ^2^ Department of Ecology, Environment and Plant SciencesStockholm UniversitySE 106 91StockholmSweden; ^3^ Department of Marine Sciences‐TjärnöUniversity of GothenburgSE 452 96StrömstadSweden

**Keywords:** asexual reproduction, clonality, macroalgae, microsatellites, somatic mutations

## Abstract

In dioecious species with both sexual and asexual reproduction, the spatial distribution of individual clones affects the potential for sexual reproduction and local adaptation. The seaweed *Fucus radicans*, endemic to the Baltic Sea, has separate sexes, but new attached thalli may also form asexually. We mapped the spatial distribution of clones (multilocus genotypes, MLGs) over macrogeographic (>500 km) and microgeographic (<100 m) scales in the Baltic Sea to assess the relationship between clonal spatial structure, sexual recruitment, and the potential for natural selection. Sexual recruitment was predominant in some areas, while in others asexual recruitment dominated. Where clones of both sexes were locally intermingled, sexual recruitment was nevertheless low. In some highly clonal populations, the sex ratio was strongly skewed due to dominance of one or a few clones of the same sex. The two largest clones (one female and one male) were distributed over 100–550 km of coast and accompanied by small and local MLGs formed by somatic mutations and differing by 1–2 mutations from the large clones. Rare sexual events, occasional long‐distance migration, and somatic mutations contribute new genotypic variation potentially available to natural selection. However, dominance of a few very large (and presumably old) clones over extensive spatial and temporal scales suggested that either these have superior traits or natural selection has only been marginally involved in the structuring of genotypes.

## Introduction

Asexual reproduction results in genetically identical offspring, but single‐locus differences can be contributed by somatic mutations (Arnaud‐Haond et al. 2007). Although only a few species are exclusively asexual, clonality has evolved repeatedly in many organism groups and is more common than expected in young (e.g., postglacial) or extreme habitats (Eckert et al. 2003; Hörandl et al. 2008; Kawecki 2008; Bengtsson 2009; Vallejo‐Marin et al. 2010). Different hypotheses have been suggested to explain the evolution of clonality as a strategy of reproduction, the most obvious one being the potentially doubled increase in population growth. Asexual reproduction is also common along the margins of species' distributions, and it has been suggested that this is a consequence of species hybridization, high physiological stress in marginal environments favouring certain genotypes (“frozen niche hypothesis”), or high costs of sexual reproduction in these habitats (Vrijenhoek 1984; Silvertown 2008; Bengtsson 2009; Vrijenhoek and Parker 2009). It has also been suggested that asexual reproduction has initial advantages during colonization of new areas where Allee effects may impede sexual reproduction (Baker 1967; Hörandl et al. 2008).

In contrast to selfing, asexual reproduction does not reduce single‐locus genetic variation of populations; instead, it conserves existing genotypes by preventing recombination of new genotypes. However, even occasional sexual activity introduces new genotypic variation into a population maintaining a high level of genotypic variation comparable to in a fully sexual population (Balloux et al. 2003; Bengtsson 2003). In populations that have potential for both asexual and sexual recruitment, the relative contribution of asexual recruitment depends both on the ratio of males to females and on the spatial distribution of individuals of separate sexes. The latter is particularly important in immobile species with restricted ranges of gamete dispersal, such as many marine seaweed species, and in these species, sexual reproduction can only take place if both males and females are present within the dispersal distance of the gametes.

In many asexual organisms, new vegetative individuals (*ramets*) are produced from the original parental individual and together they are part of the same genetic unit (the *genet*), making the primary outcome of clonal growth an increase in the size of the genet (Vallejo‐Marin et al. 2010). In macroalgae, thalli (individuals) produced by cloning are the result of adventitious branches that detach and reattach to the substratum at some distance from the parental thallus. Thus in macroalgae, ramets of the same clone (together a genet) are free‐living from each other, in contrast to clones of terrestrial plants or seagrasses that are (at least initially) connected to the parental plant by roots or rhizomes (Hämmerli and Reusch 2002; Xue‐Hua et al. 2006; Zipperle et al. 2011).

Two main spatial strategies of vegetative recruitment are common: (1) the phalanx strategy, in which aggregated structures with genetically identical ramets are clumped together forming discrete clones in which ramets of other genets are more or less excluded; and (2) the guerrilla strategy, in which genets are intermingled through a more efficient spread of ramets, resulting in clones being spatially mixed and less discrete (Lovett‐Doust 1981; Alberto et al. 2005; Ruggiero et al. 2005; Vallejo‐Marin et al. 2010). Plants in which new ramets form while still connected to the parental plant cannot disperse easily and often exhibit high levels of clonal aggregation (Reusch et al. 1999; Reusch and Boström 2011; Zipperle et al. 2011). In contrast, marine macroalgae and some aquatic plants that drop off vegetative fragments, which may be transported at least short distances by the aquatic medium, are likely to show less aggregation of single clones (Vallejo‐Marin et al. 2010).

The Baltic Sea is a truly marginal marine environment, and an enclosed estuarine ecosystem characterized by a strong and relatively permanent salinity gradient ranging from a few promille practical salinity units (PSU) to almost full oceanic salinity outside the Danish straits. In the northern parts of the Baltic Sea, the two brown macroalgal species *Fucus vesiculosus* L.*,* and *Fucus radicans* Kautsky & Bergström (Bergström et al. 2005) are the only large perennial seaweeds. *Fucus radicans* is endemic to the Baltic Sea and has a very recent (a few thousand years) origin from Baltic populations of *F. vesiculosus* (Pereyra et al. 2009, 2013). Both species have separate sexes and recruit new thalli by zygote formation of short‐lived gametes. Both species are also capable of producing new attached thalli by fragmentation (Tatarenkov et al. 2005; Johannesson et al. 2011). Asexual reproduction in these species occurs by dropping vegetative fragments (adventitious branches) that reattach to the substratum and grow into new fully fertile thalli (Bergström et al. 2005; Tatarenkov et al. 2005).

The mixed mode of reproduction will influence the spatial genetic structure, and at small spatial scales, this will affect the opportunities for sexual reproduction of individual plants and thus the species' potential to evolve local adaptation by forming new genotypes from recombination of existing ones (Reusch et al. 1999; Epperson and Chung 2001; Charpentier 2002; Vekemans and Hardy 2004; Ruggiero et al. 2005; Pereyra et al. 2013). At a macrogeographic scale, the distribution of individual clones may affect the genetic structure of populations, at least when populations contain few clones, and contribute to skewed sex ratios in dioecious species and potential problems to recruit sexually.

Here, we investigated the detailed (microscale) spatial distribution of clones identified from microsatellite genotypes in the dioecious seaweed *Fucus radicans* in part of its distribution where it is highly clonal. We asked the question whether clones are distributed in a phalanx‐ or a guerrilla‐like pattern, and our prediction was that in contrast to seagrasses that show a phalanx distribution of clones (Hämmerli and Reusch 2002; Zipperle et al. 2011), *F. radicans* would have a more intermingled configuration of genotypes and sexes similar to other fragment dispersed organisms (e.g., mosses, lichens, and sponges) (Wulff 1991; Heinken 1999; Cleavitt 2002). We used spatial autocorrelation analysis of local genotype distribution to test this prediction. In a dioecious species, a guerrilla‐like pattern of distribution will promote sexual recruitment, and we assessed the rate of sexual recruitment by looking for individuals of genotypes that were clearly separated from genotypes of dominant clones.

We furthermore mapped the macrogeographic distribution of clones over all 16 populations to find out whether clones were generally local or widespread. Using the microsatellite information, we also inferred which clones (MLGs) were likely of close ancestry (genotypes differing by 1–2 mutations only) and considered these to be members of the same multiclonal lineage (MLL) (Arnaud‐Haond et al. 2007). We finally asked the question how important were sexual versus asexual recruitment of new MLGs into the population, and whether populations with highly intermingled clones of different sex had higher sexual recruitment than populations dominated by one sex.

## Materials and Methods

**Figure 1:**
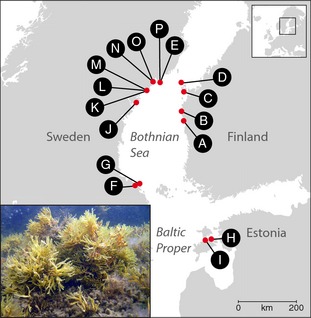
Geographic positions of the 16 analyzed populations of *Fucus radicans*, and an illustration of *Fucus radicans*.

In this study, we analyzed data from 16 populations of the brown algae *F. radicans* (Fig. 1). Ten of these were previously studied in Johannesson et al. (2011), three additional populations were analyzed at the population level (Swe K, Swe L, and Swe M), and another three were analyzed not only at population level but also at a microscale level (Swe N, Swe O, and Swe P) (Table 1). The 16 populations span the species' northern–southern distribution (the Bothnian Sea and the Estonian coast of the Baltic Proper), while potential populations in the Gulf of Finland were not included in this study (Fig. 1).

**Table 1:** Populations of *Fucus radicans* included in the macrogeographic analyses

Sampling site	Region	Population	Coordinates	Ramets	Genets	*H*_*O*_	*H*_*E*_	Sampling year	Reference
Sälskär	Finland	Fin A	62°19′N, 21°10′E	29	29	0.54	0.57	2007	Johannesson et al. (2011)
Märigrund	Finland	Fin B	62°31′N, 21°03′E	45	33	0.54	0.58	2007	Johannesson et al. (2011)
S. Vallgrund	Finland	Fin C	63°09′N, 21°13′E	50	5	0.56	0.53	2007	Johannesson et al. (2011)
Hällkalla	Finland	Fin D	63°25′N, 20°57′E	50	12	0.37	0.34	2007	Johannesson et al. (2011)
Järnäs	Sweden	Swe E	63°26′N, 19°40′E	47	13	0.52	0.48	2003	Johannesson et al. (2011)
Öregrund	Sweden	Swe F	60°22′N, 18°21′E	48	13	0.64	0.46	2003	Johannesson et al. (2011)
Djursten	Sweden	Swe G	60°23′N, 18°24′E	48	16	0.67	0.49	2007	Johannesson et al. (2011)
Pulli panki	Estonia	Est H	58°36′N, 22°58′E	13	13	0.52	0.61	2006	Johannesson et al. (2011)
Triigi	Estonia	Est I	58°35′N, 22°43′E	6	6	0.53	0.62	2006	Johannesson et al. (2011)
Bönhamn	Sweden	Swe J	62°53′N, 18°18′E	30	5	0.64	0.51	2007	Johannesson et al. (2011)
Skagsudde	Sweden	Swe K	63°11′N, 19°01′E	47	9	0.51	0.44	2010	This study
Skagsudde	Sweden	Swe L	63°11′N, 19°00′E	60	17	0.66	0.58	2007	This study
Skagsudde	Sweden	Swe M	63°11′N, 19°00′E	57	10	0.42	0.51	2012	This study
Drivan	Sweden	Swe N	63°26′N, 19°20′E	60	7	0.46	0.39	2010	This study
Drivan	Sweden	Swe O	63°26′N, 19°20′E	79	10	0.49	0.44	2010	This study
Järnäs	Sweden	Swe P	63°26′N, 19°40′E	59	8	0.68	0.45	2010	This study

Number of ramets *(n)*, genets (multilocus genotypes, MLGs), average observed heterozygosity (*H*_*O*_), and average expected heterozygosity (*H*_*E*_) in 16 population of *Fucus radicans*.

### Study populations

For the microscale study, tissue from *F. radicans* thalli was collected (July 2010) from three populations (Swe N, Swe O, and Swe P) close to the northern distributional limit of the species along the Swedish coast of the Bothnian Sea (Fig. 1). In each population, we sampled three 2‐m‐wide corridors along 30‐m‐long measuring tapes within the *Fucus* belt, using a common point of origin for the separate corridors spread in different directions. A small branch of thalli with receptacles and vegetative tips was picked from individuals of *F. radicans* within each corridor. Each branch was tagged and placed in a bag for determination of sex and stored for genetic analysis. The diver accounted the relative position of each thallus by noting the depth, distance from the tape (using a smaller handheld measuring tape), and distance along the tape for each sampled thallus. Samples were taken from between 0.5 and 6.0 m depth at three different sites; Swe N and Swe O were separated by 135 m, and Swe P was 20.1 km from the two others. For this microgeographic analysis, a total of 198 thalli were analyzed: 60 from Swe N, 79 from Swe O, and 59 from Swe P.

In the macrogeographic analysis, 13 additional populations were analyzed including samples from northern Finland (Bothnian Sea), eastern Sweden (Bothnian Sea), and Estonia (Baltic Proper) (see Fig. 1 and Table 1).

### DNA extraction and microsatellite genotyping

Tissue samples (5–6 cm per thallus) were mechanically cleaned of epiphytes to reduce contamination and stored separately in plastic bags with silica gel to avoid DNA degradation. Dried tissue was pulverized in a milling instrument. DNA was extracted using the NucleoSpin^®^ Plant II kit (MACHEREY‐NAGEL, Düren, Germany) according to standard kit instructions. Samples were amplified and genotyped at nine microsatellite loci (L20, L38, L58, L85, and L94: Engel et al. 2003; and Fsp1, Fsp2, Fsp3, and Fsp4: Perrin et al. 2007) as in Johannesson et al. (2011). Fragments were sized and analyzed on a capillary sequencer (CEQ8000; Beckman Coulter Inc., Fullerton, CA).

### Data analysis

The new sets of microsatellite data (six populations; see Table 1) were checked for null alleles, stuttering, genotypic errors, and large allelic dropout by 1000 randomizations using the MICRO‐CHECKER v. 2.2.3 (van Oosterhout et al. 2004). For the genet data of all populations, we used GENEPOP 4.2 (Rousset 2008) to generate allele frequencies, observed and expected heterozygosities (*H*_O_ and *H*_E_), deviations from Hardy–Weinberg equilibrium (HWE), and linkage disequilibrium (LD).

### Analysis of microgeographic structure

#### Clonality and spatial analyses

We used spatial autocorrelation analysis (Sokal and Wartenberg 1983; Smouse and Peakall 1999) to study the effects of fragment dispersal and spatial genetic structure in three of the populations. This has previously been performed in terrestrial plant populations (Epperson and Chung 2001; Vekemans and Hardy 2004; Gapare and Aitken 2005; Roe et al. 2011) and also in marine seagrasses (Reusch et al. 1999; Ruggiero et al. 2005; Zipperle et al. 2011). The method examines the genetic relatedness between pairs of individuals with regard to their relative positions in space (Alberto et al. 2005) and estimates clonal subranges (local areas of distribution of the same clone) and spatial scales over which clonal processes still appear to affect the local genetic structure of the population.

GENCLONE 2.0 (Arnaud‐Haond et al. 2007) is designed for studying clonality and its spatial components using genotype data with molecular markers from haploid or diploid organisms. Using the relative plot coordinates for every sampled *F. radicans* thallus, autocorrelation analyses were performed with this software at both genet and ramet level. The spatial autocorrelation analysis represents the degree to which a set of spatial coordinates and their associated genetic data values tend to cluster together in space (positive spatial autocorrelation) or disperse (negative spatial autocorrelation) (Loiselle et al. 1995; Ritland 1996; Epperson and Li 1997; Rousset 2000; Arnaud‐Haond et al. 2007). Information of the spatial coordinates of the sampled thalli allowed for determining whether clones (and thereby also sexes) occurred aggregated or intermingled in the population. Combining the spatial coordinates for each thallus together with the thallus' genotype data, a geographic map of the spatial distribution of multilocus genotypes (MLGs), multilocus lineages (MLLs), and sexes were obtained for each of the three populations. To further determine the extent of spatial aggregation of genotypes, we estimated the aggregation index *A*_*c*_. The clonal subrange for every MLG found in each site was also estimated in GENCLONE 2.0 as the largest geographic distance between sampling units sharing the same MLG (Alberto et al. 2005; Arnaud‐Haond et al. 2007). A test for an edge effect *E*_*e*_ was also performed, that is, if large clones are represented fewer times than expected because positioned at the edge of the transect.

MLGs can be a result of either sampling the same clone at two different spatial coordinates (different ramets of the same genet), or from sampling two thallus of the same genotype originating from two distinct sexual events. To separate these hypotheses, we determined the probability that thalli with identical genotypes derived from separate sexual reproductive events (*P*_sex_). Tests were also performed to check whether the number of sampled units (*n*) per area gave a good estimate of the clonal diversity of the area sampled. Alternative methods were used to approximate clonal diversity within the three localities such as genotypic richness, *R* = (G−1)/(*N*−1), where G is the number of multilocus genotypes and *N* is the sample size (Dorken and Eckert 2001), representing the absolute number or the proportion of distinct clonal lineages or genets present in the sample, comparative to the number of sampling units. The Pareto distribution model (Vidondo et al. 1997; Arnaud‐Haond et al. 2007) was used to describe the frequency distribution of clonal membership for the microgeographic localities of *F. radicans*. A high evenness with clonal lineages having all approximately comparable numbers of ramets will result in a steep slope in a Pareto plot, whereas a skewed distribution with very few, large clonal lineages containing the majority of ramets will result in a shallow slope.

### Analysis of macrogeographic structure

To view the spatial genetic structure and clonal distribution at a wider geographic range, analyses were carried out where the three earlier described populations were supplemented with new data from three additional populations: Swe K, L, and M, sampled 2007, 2011, and 2012, respectively (K and L were separated by 1100 m, L and M by 700 m, and K and M by 400 m), and with previously published data from 10 localities across the known distributional range of *F. radicans* in the Baltic Sea (Johannesson et al. 2011), resulting in a total data set of 16 populations (Table 1). Three populations were samples in close proximity to each other (Swe N and O [locality Drivan], and Swe K, L, and M [locality Skagsudde]) as described above. Two samples (Swe E and P) were sampled at the same coordinates, but 7 years apart, and for simplicity, we also refer to these samples as different “populations.”

#### Population genetic statistics and structure

A matrix of pairwise estimates of between‐population divergence (*F*_ST_) on genet level was obtained from GENEPOP. Using the web‐based program IDBWS (Jensen et al. 2005), a Mantel test with 10,000 random permutations was performed between a pairwise geographic (linear) matrix and the genetic distance matrix using all 16 populations, to assess the effects of isolation by distance (Wright 1943). A Bayesian clustering analysis implemented in STRUCTURE 2.3.3 (Pritchard et al. 2000) was used to assign thalli to genetic clusters and define the optimum number of subpopulations (*K*). We used two different datasets: one including all genets from all 16 localities, and one in which we removed the four populations that differed the most (the two from Estonia and two southern Finnish populations) to increase the power of resolution among the remaining 12 populations. The optimal number of subpopulations was calculated for each analysis, where the *K* with the highest probability was indicated by the lowest Pr(*X*¦*K*) value (Pritchard et al. 2000).

#### Origins of clones and somatic mutations

GENCLONE 2.0 was used to assign each thallus to a certain MLG and estimate the probability that each MLG was a true clone consisting of thalli originating from the same sexual event (*P*_sex_). Multilocus lineages (MLLs) were manually created by grouping MLGs that differed by one or two mutations from a central (large) MLG, or differed by maximum one mutation from an MLG already connected to the central MLG. Also singletons (MLGs represented in the samples by only one thallus) that differed by 1 or 2 mutations from a clone were considered when building MLLs.

## Results

### Genetic variation within populations

Six of the populations included in this study were surveyed for the first time and showed similar levels of single‐locus variation (*H*_O_ and *H*_E_) as in populations surveyed earlier in this area of the species' distribution (Johannesson et al. 2011) (see Fig. 1 and Table 1). In genets from the six new populations (ramets excluded), we found no evidence of null alleles, and no evidence of scoring errors from large allele dropout or stuttering at any of the nine microsatellite loci. No pairs of microsatellite loci were significantly linked across all samples (Bonferroni‐corrected tests on genets only). Thus, the nine scored loci were all polymorphic and considered independent.

A total of 36 alleles were identified in the six new localities, and the average number of alleles per locus was 1.3–4.5, ranging from 8 (*Fsp*2) to 1 (*L*94). Three loci (Fsp3, Fsp4, and L85) showed significant (*P *<* *0.05) departures from Hardy–Weinberg equilibrium (HWE) in all six localities using GENEPOP 4.2. In Swe P, four additional loci (Fsp2, L20, L38, and L58) also deviated from HWE. Most loci showed a significant heterozygote excess, but in locus Fsp3 the deviation from Hardy–Weinberg was due to a heterozygote deficiency in Swe N and Swe O. Overall, results were similar when running separate analyses excluding different loci one by one.

### Microgeographic structure

**Figure 2:**
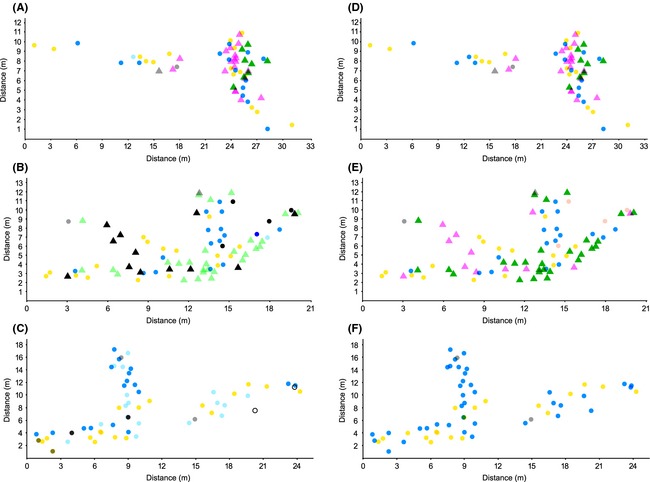
Microgeographic distribution of multilocus genotypes (MLGs; A–C) and multilocus lineages (MLLs; see text for definition; D–F) in two geographic dimensions (x and y with axes indicating distances in meters from an arbitrary reference point 0,0) in three Swedish localities, N, O, and P (see Table 1 and Fig. 1). The sex of each individual is indicated (circle – female; triangle – male), while color represents distinct MLGs and MLLs, respectively. Note that the same colors are used also in Figures 4 and 5, with white and black indicating clones that exist only in a local site and gray indicating unique MLGs (singletons).

**Figure 3:**
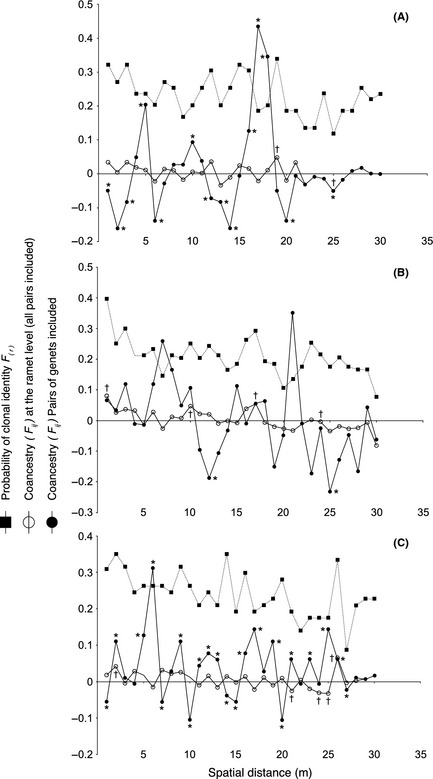
Spatial autocorrelation analysis of kinship coefficients for *F. radicans* in populations Swe N (A), Swe O (B), and Swe P (C). Each of the three correlograms shows both a ramet‐level analysis which includes all pairs sampled (coancestry *F*_(*ij*)_ at the ramet level), a genet‐level analysis with only pairs of genets included (coancestry *F*_(*ij*)_ at the genet level), and the probability of clonal identity, *F*_(*r*)_, which estimates the clonal subrange, all on the *y*‐axis. * and † indicate significant *P‐*values for genet and ramet level, respectively.

In the three northern populations that we assessed for microgeographic clonal structure, most thalli belonged to large clones, as indicated by shallow slopes of the Pareto distribution (Fig. S3A–C). Two female clones were common in all three populations (blue and yellow, Fig. 2A–C). Different male clones were found in two of the local populations (pink, and dark and light green), while in the third site males were absent (Fig. 2A–C). Spatial aggregation of MLGs (*A*_c_) was generally low (0–0.2), although significant in two of the three populations (Table 2), which supports a more intermingled distribution of genotypes. One of the localities (Swe O) showed a significant edge effect (*E*_*e*_)*,* which may result in clonal diversity being somewhat overestimated (Table 2). The probability that two thalli were members of the same clone decreased slightly with increasing geographic distance (Fig. 3); for example, thalli <1 m from each other showed a 31–39% probability of clonal identity, compared to 8–24% at 30 m distance, in the three study populations. On average, clonal subranges extended far beyond the sampling area in all three localities (Fig. 3), suggesting large spatial distributions of most of the clones. The ramet‐level spatial autocorrelation analysis rendered a few significant kinship coefficients, and these were positively correlated for shorter distances (clustered) and negatively correlated for larger distance classes (dispersed) (Fig. 3). Most coefficients were nonsignificant, and this suggested an overall random mixing of ramets of different degrees of kinship. Also the genet‐level spatial autocorrelation analyses showed several of the kinship coefficients being significant, but the positive and negative values were spread among distance classes (Fig. 3), suggesting that the microgeographic genetic structure of populations was essentially independent of the kinship relationship among genets. Thus, overall, pairs of ramets, or pairs of genets, that were genetically similar were not more spatially associated in the populations than other pairs.

**Table 2:** Genotypic richness and clonal aggregation in *Fucus radicans*

Site	R (genotypic richness)	Aggregation index (*A*_c_)	Edge effect (*E*_E_)
Swe N	0.17	0.00	−0.69
Swe O	0.11	0.12^a^	0.29^a^
Swe P	0.14	0.20^a^	0.17

Indexes used to describe the genotypic richness, clonal aggregation, and edge effects at microgeographic scale in three populations of *Fucus radicans*.

a Indicates significant *P‐*values (α < 0.05).

In conclusion, the overall microgeographic genetic structure of *F. radicans* was in all three study populations characterized by a high level of intermingling of ramets of a few dominant clones with additional scattered thalli of small clones. From the spatial autocorrelation analysis, it was also obvious that many clones had extensive distributional ranges. With respect to gender, two of the three populations were made up by dominant clones of different sex, while in the third population, the two dominant clones were both females and no male was found.

### Macrogeographic structure

#### Population genetic structure

A majority of the study populations were genetically different in pairwise comparisons (see *F*_*ST*_ matrix*,* Table S1), and overall, there was an isolation‐by‐distance effect at the macrogeographic scale (Mantel test, *P *<* *0.0001; Fig. S1A, and *P *<* *0.001; Fig. S1B), although mostly driven by differences at the largest spatial scale (countries). The population structure analysis showed that a division into *K* = 6 clusters was most strongly supported (highest Pr(*X*¦*K*) value); in addition, some localities in close proximity differed substantially from each other (Fig. S2A, comparing Swe M with Swe K and Swe L), while the opposite was also sometimes true (Fig. S2A, comparing Swe M with Fin C, and Fin D). When we reanalyzed the weakly differentiated populations separately (excluding the two southernmost Finnish populations and the two Estonian ones), *K* = 3 clusters were instead supported (Fig. S2B). This indicated that there were three genetic groups present in the north and west Bothnian Sea and that all 12 populations contained components of 2–3 of these groups (Fig. S2B). (These analyses were carried out on genet variation, and dominance of single clones could not explain these patterns.)

#### Clonal distribution

**Figure 4:**
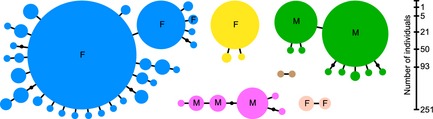
Network of multilocus genotypes (MLGs) showing multilocus lineages (MLLs). Each circle represents a separate MLG, and each color indicates a separate MLL (colors as in Figs 2 and 5). Circle size is proportional to numbers of individuals, and figures on scale reflect numbers of individuals of the largest MLG of each MLL. When present, letters indicate that sex was confirmed (F for female, M for male). MLGs are clustered into MLL by joining those MLGs that differ by 1–2 mutations.

**Figure 5:**
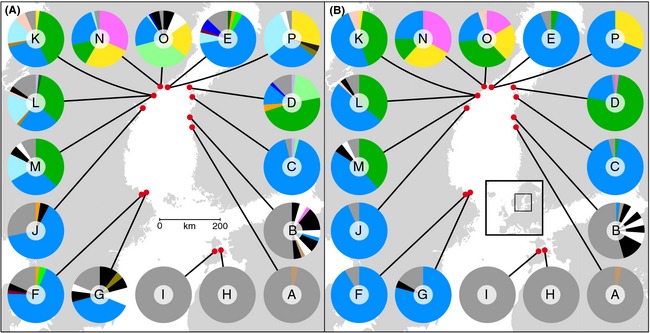
Macrogeographic distribution of singletons (gray), local clones (black and white), and widespread clones (different colors) in the Baltic Sea (A). In (B), MLGs that differ with 1–2 mutations (see Fig. 3) are merged into multilocus lineages (MLLs). For details on sample sizes and positions, see Figures 1 and Table 1.

The probability values for MLGs being of different sexual origins were significantly low (*P*_sex_ **<* *0.05), and it was therefore concluded that all ramets with identical MLG shared a common origin and belonged to the same clone. Moreover, many of the small, local clones and individuals of unique MLGs (singletons) differed by 1–2 somatic mutations from a larger clone and were identified as members of large clonal lineages (MLLs; see Materials and Methods) (Fig. 4). We found four large clonal lineages, and two smaller. From sexing of several thalli within each clonal lineage, we found that three of them were females (blue, yellow, and light pink MLL) and two were males (green and pink). The clonal lineage that was the largest (blue female) also had the highest number of genotypes in the MLL network (30), and in addition to the central MLG, there was one other common and widespread MLG in this network (light blue in Figs 2C and 5A). The other three large clones had less complex networks of MLGs associated, but the large male clone (green) had a relatively common second MLG (light green, Figs 2B and 5A).

The large clones and the four large clonal lineages dominated much of the Bothnian Sea distribution of *F. radicans* (Fig. 5A–B). Two of the lineages, the blue female and the green male lineages, co‐occurred in many of the northern populations, while the blue female lineage alone tended to dominate populations on both the west and east coast of the Bothnian Sea (Fin C, and Swe G, F, and J). The distribution of the blue female was in many ways exceptional, being common in 12 of 16 populations and distributed over a distance of 550 km. The large male clonal lineage (green) was also extensively distributed, present in 8 of 16 populations, and spread over 100 km of coastline in Sweden and Finland. Two additional clones (pink male and yellow female) were locally dominant but only found in adjacent localities (Swe N, O, and P; Fig. 4B). Two minor clones were also found in more than one site, but only represented by a few thalli each (light pink and brown in Fig. 5B). Notably, the genotypes of both the yellow clonal lineage and the light pink clonal lineage suggested these were offspring of a cross between the blue female and the green male. Local clones that were more distantly related than a few mutations to any of the dominant clonal lineages were found in some populations (indicated by black and white sectors in Fig. 5A–B). One population, in particular, stood out with 50% of the thalli being members of small and local clones (Fin B). This population was on the border between northern Finnish populations dominated by the large clonal lineages, and a southern site (Fin A) that was almost completely dominated by unique MLGs distinct from the large clonal lineages and most likely of recent sexual origins. Also the two Estonian populations seemed to be completely sexually recruited. This was in sharp contrast to northern and western populations where numbers of unique MLGs not linked to any clonal lineage and of putatively recent sexual origin were 0–8 (average 2.5) per population (gray in Fig. 5B).

## Discussion

Our result evidences a highly complex geographic structure of clones in *Fucus radicans*. We found both a few very large, widespread and presumably old clones, and many small, local and probably much younger clones, many of which were mutants of the large dominant clones (Fig. 4). In addition, we found MLGs only represented by one individual (singletons). The singletons most different to other clones were likely to be sexually recruited or copies of small new clones recently established by sexual recruitment, while others only differed by 1–2 mutations from the dominant clones and where likely local mutants. While several populations in the north were dominated by 1–4 of the large clones and clonal lineages, the most southern populations were predominantly sexually recruited. One population stood out having a mix of recruited singletons and small local clones, and this population was situated in between the typical asexual and typical sexual populations. On a local scale, we found that in populations dominated by a few large clones, these were intermingled in a guerrilla‐like distribution. Despite the potential for efficient gamete interaction, these populations were highly asexual. One explanation may be that when asexual recruitment becomes dominant, this may be associated with variable sex ratios that may affect sexual efficiency. Indeed, we here found that some of the strongly clonal populations had skewed sex ratios, with the most extreme example having only female genets (Fig. 2), and this may have contributed to low sexual recruitment in some areas.

While many asexual populations have only local clones due to vegetative reproduction through rhizomes or roots such as in plants (Eriksson 1993; Reusch and Boström 2011), or fragments without dispersal capacity, such as in stony corals (Dahl et al. 2012), we found several of the *F. radicans* clones to have extensive geographic distributions. Indeed, species that reproduce asexually by fragmentation or other vegetative parts that may spread long distances (e.g., seaweeds, mosses, lichens, and marine sponges, but also some aquatic plants) will more likely include widespread individuals of the same clone (Wulff 1991; Heinken 1999; Cleavitt 2002).

Although there is a potential for spread of clones, only a minor proportion (4–5 of >30) of the clones were geographically spread, and of these, the large female clone was outstanding. The many local mutant clones derived from this clone strongly suggest that this is a very old clone. It seems indeed likely that much of the species' distribution along the Swedish and northern Finnish coasts of the Bothnian Sea (Forslund et al. 2012) consists of individuals of the female clone. With densities of thalli in the range of 10 per m^2^ or more, the number of ramets of this species must be millions or tens of millions. To reach such a large size and establish over such a large geographic area, this clone likely originated early in the history of this young species (Pereyra et al. 2013) and may thus be some few thousand years old. The large male clone, as well, has a distribution over 100 km of coastline and is also associated with a number of mutated genotypes. This male clone may also be rather old. The yellow clone that has a genotype that is compatible with a cross of the large female and male clone plus one mutation is locally common, but has a much more restricted distribution and only few mutated varieties, and is likely to be considerably younger than the other two clones. The pink clone, on the other hand, had several almost similarly sized genotypes in its network and may be relatively old. Thus, at least a few of the large clones seem likely to be not only extensively distributed but also relatively old.

From what we observed in the sampled populations, almost all adult thalli form receptacles and gametes. Furthermore, numbers of egg produced per receptaculum in the large female clone are similar to numbers produced in mainly sexual populations of Baltic Sea *F. vesiculosus* (Forslund and Kautsky 2013). With sexually active individuals and clones of both sex thoroughly mixed, gamete interactions and zygote formation should be possible (Serrão et al. 1997). As mentioned above, the genotype of the yellow clone also suggests that sexual recombination may occur. However, rather low proportions of the genotypes in the northern populations were singletons (gray sectors in Fig. 5A) and even fewer were not part of any large clonal lineage (gray sectors in Fig. 5B). Thus, sexual activity seems strongly constrained, despite the mixed distribution of male and female clones in most northern populations. Earlier studies have shown that low salinity restricts successful fertilization due to, for example, lysis of the egg cell or polyspermy (Serrão et al. 1999a), and this may contribute to the low sexual recruitment in the northern Swedish and Finnish populations where salinity is low (3.5–4.3 PSU; salinity data from Johannesson et al. 2011), but at the same time, populations on the Finnish side (A and B), as well as in Estonia (H and I), showed high sexual activity despite only slightly higher salinities (5.2–5.8 PSU). Indeed, earlier studies show a dramatic decrease in egg fertility somewhere between 4 and 6 PSU (Serrão et al. 1996a), and thus, a shift from asexual to sexual recruitment may happen somewhere in this range of salinities. Notably, the southernmost populations sampled along the Swedish coast (Swe F and Swe G) were again dominated by the large female clone, but had a minor proportion of singletons different from the large female clonal lineage that may indicate some sexual activity at a relatively high salinity (5.0 PSU). In these populations, a strong female‐biased sex ratio may also contribute to impede sexual recruitment.

The Finnish population B stood out compared to all other populations as having equally high frequencies of both asexual and sexual recruitment. In Fin B, none of the four large clonal lineages were common; instead, small local clones and unique singletons of recent sexual ancestry made up the majority of the population. This site is 20 km north of the populations of the Finnish A population with a nearly completely sexual population and 60 km south of the site of Fin C that has almost no sexual activity. This suggests, perhaps, that this a transient area for a shift from asexual to sexual reproduction imposed either by a salinity shift or by some yet unknown features.

Phenotypic variation among clones of a species may be extensive, and the basis for local adaptation in asexual populations (e.g., Achenbach et al. 2012), although selection is expected to be much slower in the absence of recombination. Clones of *F. radicans* differ in inherited traits, such as tolerance to desiccation and freezing, and tolerance to grazing (as indicated by differences in phlorotannin content) (Johannesson et al. 2012). Thus, there is a basis for selection among clones, and opportunities for local adaptation of populations. Hence, it is surprising that in *F. radicans*, the same few clones are established over temperature, salinity, and grazing gradients spanning from northern to southern Bothnian Sea and further into the Baltic Proper. For example, temperatures and salinities are generally lower in the northern parts of the species' distribution (Liu et al. 2013), while fucoid grazers are much less abundant in the north than in the south (Leidenberger et al. 2012). Given a high age of the largest clones, it is also notable that these clones have survived substantial climatic changes over the past thousands of years in the postglacial Baltic Sea (Zillén et al. 2008). What explains the extensive temporal and spatial distribution of the large clones? A possible hypothesis is that these clones have superior generalist phenotypes that resist a range of different environments, and/or have higher capacity of dispersing and recruiting than other phenotypes. Alternatively, stochastic demographic processes during colonization and establishment of the new species may result in a few genotypes being much more numerous than others (e.g., Waters et al. 2013).

Perhaps, stochasticity is also introducing substantial variation at the microgeographic scale. We repeated sampling after 7 years in one location (Swe E and P) and this showed an obvious change in the composition of genotypes. One MLL (yellow female) not present at all in the first sampling had a frequency of 30% in the second sampling (Fig. 5). Indeed, one other location (Swe N) had an even sex ratio at the time of our sampling 2010 (Fig. 5), but sampled in 1996 it had a sex ratio that was highly skewed toward females (80%, *n* = 82, Serrão et al. 1999b).

The potential for long‐distance dispersal is a key component whether or not selection among clones or random events is crucial in structuring the geographic pattern of clones. With long‐distance dispersal, a clone present in one area is able to spread and establish in a distant area, and a unisexual population may turn into a bisexual population and initiate sexual reproduction. In *F. radicans* gametes, zygotes, adventitious branches, and thalli all have negative buoyancy (Serrão et al. 1996b; D. Johansson, pers. obs.) and long‐distance dispersal seems much more rare than in the close relative *F. vesiculosus* that has floating bladders. Some indirect observations, however, show occasional long‐distance dispersal also in *F. radicans*. A single large thallus of an attached *F. radicans* was found 50 km northeast of the nearest population at the border of the species' northern distribution (Länsstyrelsen, 2008), and a single drifted (fully vital) thallus was found in a shore 18 km north of the same populations (R. Pereyra, pers. obs.). In the present study, we also found single thalli that genetically assigned to populations from distant sites, rather than to the population in which they were found. In the Swe F population, as the most clear‐cut example, there was one thallus that assigned to the Estonian populations with a very high probability (Fig. S2A–B). However, under the dominance of a few large clones, also long‐range dispersal may mostly be a transport of the same dominant genotypes among populations where they are already established.

In conclusion, the Baltic endemic seaweed *Fucus radicans* seems to offer a number of intriguing features worthy of further exploration. For example, the reasons for the wide variation in asexuality and the dominance in much of the species' distribution of a few extensively large clones need comprehensive investigations. Moreover, there is a potential to use genomewide markers to address issues of within‐clone evolution and components of adaptive evolution along spatial gradients. To further understand the evolution of reproductive polymorphism, ecological experiments should be used to investigate the allocation of reproductive efforts into sexual and asexual strategies and the potentially obstructing role of salinity for gamete functionality. Finally, investigations of the role of stochastic demographic events in the colonization history of the species may throw additional light on the complex spatial genetic structure of the species.

## Conflict of Interest

None declared.

## Supporting information


**Figure S1** A‐B. Mantel tests for matrix correlation between genetic distance (*F*_*ST*_) and geographic distance (km). Scatter plot A shows pairwise *F*_*ST*_ versus geographic distances of 16 localities of *F. radicans* in the Baltic Sea showing significant correlation between geographic and genetic distance (*P* = 0.0001). Scatter plot B shows the pairwise *F*_*ST*_ versus geographic distances without the two Estonian populations (*P* = 0.001).
**Figure S2** A‐B. STRUCTURE analyses of nine microsatellite genotypes of genets from 16 sites (Fig S2 a) resp. 12 sites (Fig S2 b) of *F. radicans* in the Baltic Sea. Each vertical bar represents one individual. Fraction of color in an individual represents its estimated membership to a certain genetic group. Black lines with arrows separate the different localities. Sampling sites are labelled below the figure and region labelled above. K (number of groups) is 6 for S2 a, and 3 for S2 b, which was supported by values of Pr(X¦K).
**Figure S3** A‐C. The Pareto distribution analysis describes the frequency distribution of clonal membership for the micro‐geographic localities of *F. radicans*. A skewed distribution with very few, large clonal lineages containing the majority of ramets, will result in a shallow slope, as seen here.
**Table S1** Pairwise *F*_*ST*_‐values of *F. radicans*.
