# Supplementary material for: Complex spatial clonal structure in the macroalgae Fucus radicans with both sexual and asexual recruitment
Source: Ecol Evol. 2015 Sep 9;5(19):4233–45. doi: 10.1002/ece3.1629 (PMC4667831; doi:10.1002/ece3.1629)
Supplement: Supplementary file 1 — Figure S1 A‐B. Mantel tests for matrix correlation between genetic distance (F ST) and geographic distance (km). Scatter plot A shows pairwise F ST versus geographic distances of 16 localities of F. radicans in the Baltic Sea showing significant correlation between geographic and genetic distance (P = 0.0001). Scatter plot B shows the pairwise F ST versus geographic distances without the two Estonian populations (P = 0.001). Figure S2 A‐B. STRUCTURE analyses of nine microsatellite genotypes of genets from 16 sites (Fig S2 a) resp. 12 sites (Fig S2 b) of F. radicans in the Baltic Sea. Each vertical bar represents one individual. Fraction of color in an individual represents its estimated membership to a certain genetic group. Black lines with arrows separate the different localities. Sampling sites are labelled below the figure and region labelled above. K (number of groups) is 6 for S2 a, and 3 for S2 b, which was supported by values of Pr(X¦K). Figure S3 A‐C. The Pareto distribution analysis describes the frequency distribution of clonal membership for the micro‐geographic localities of F. radicans. A skewed distribution with very few, large clonal lineages containing the majority of ramets, will result in a shallow slope, as seen here. Table S1 Pairwise F ST‐values of F. radicans. [file ECE3-5-4233-s001.docx]

**Supplementary Information**

**Figures**

**Figure S1 A-B.** Mantel tests for matrix correlation between genetic distance (*F_ST_*) and geographic distance (km). Scatter plot A shows pairwise *F_ST_* vs. geographic distances of 16 localities of *F. radicans* in the Baltic Sea showing significant correlation between geographic and genetic distance (*P* = 0.0001). Scatter plot B shows the pairwise *F_ST_* vs. geographic distances without the two Estonian populations (*P* = 0.001).

**Figure S2 A-B.** STRUCTURE analyses of nine microsatellite genotypes of genets from 16 sites (Fig S2 a) resp. 12 sites (Fig S2 b) of *F. radicans* in the Baltic Sea. Each vertical bar represents one individual. Fraction of color in an individual represents its estimated membership to a certain genetic group. Black lines with arrows separate the different localities. Sampling sites are labelled below the figure and region labelled above. K (number of groups) is 6 for S2 a, and 3 for S2 b, which was supported by values of Pr(X|K).

**Figure S3 A-C.** The Pareto distribution analysis describes the frequency distribution of clonal membership for the micro-geographic localities of *F. radicans*. A skewed distribution with very few, large clonal lineages containing the majority of ramets, will result in a shallow slope, as seen here.

**
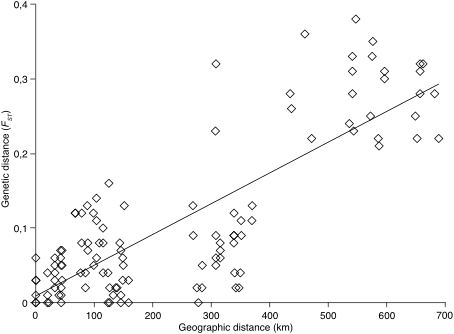
**

**Fig. S1 A**

**
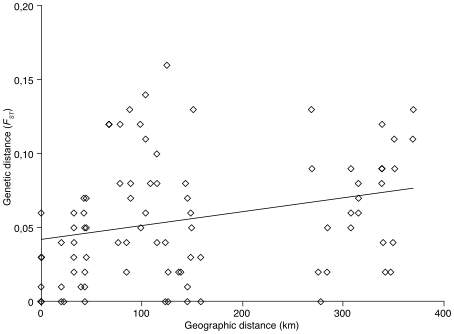
**

**Fig. S1 B**


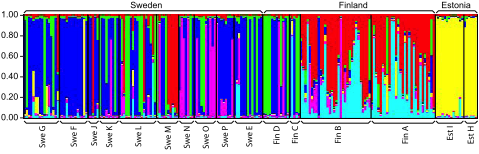


**Fig. S2 A**


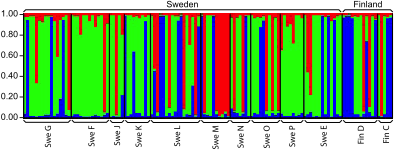


**Fig. S2 B**


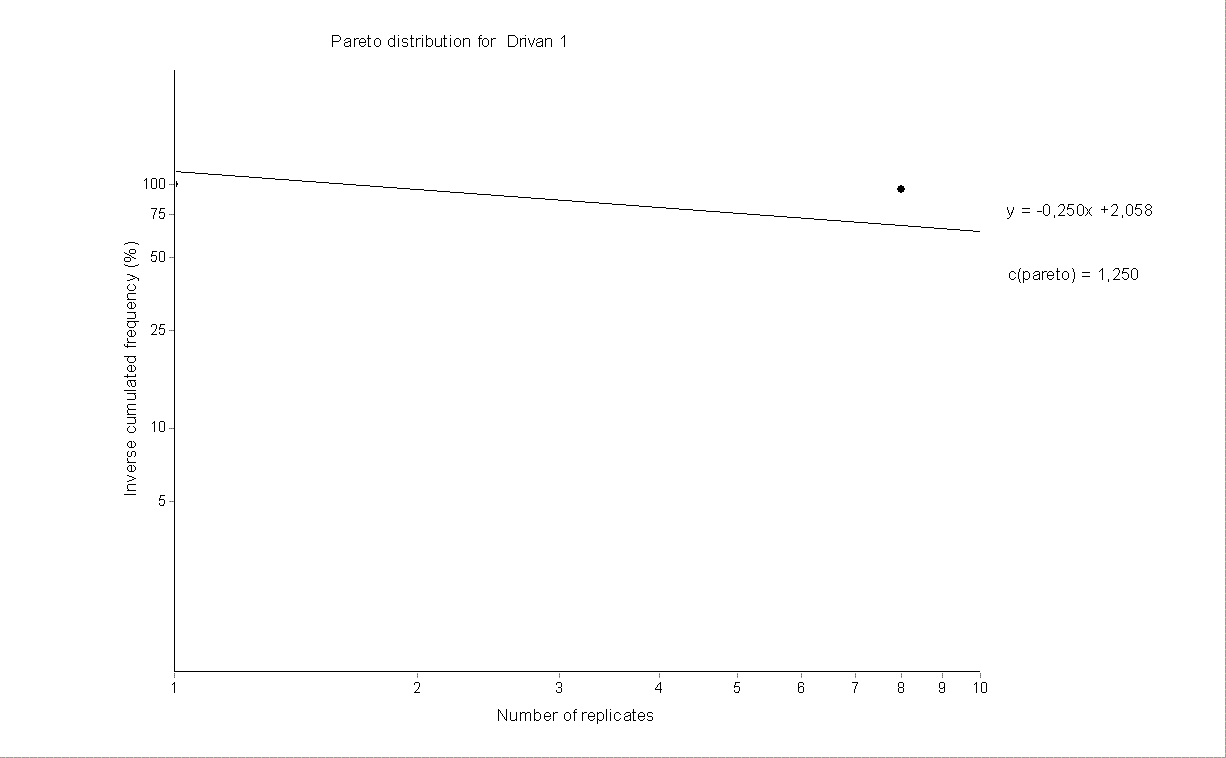


**Fig. S3 A**


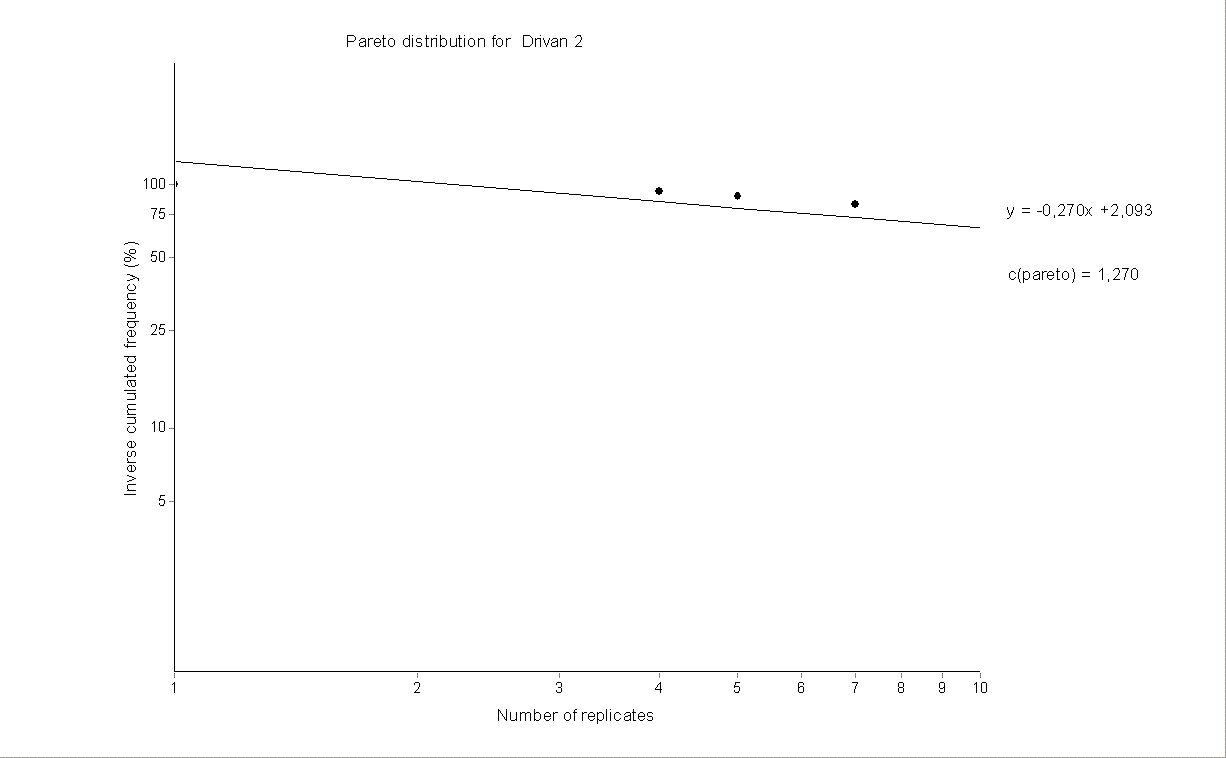


**Fig. S3 B**

**
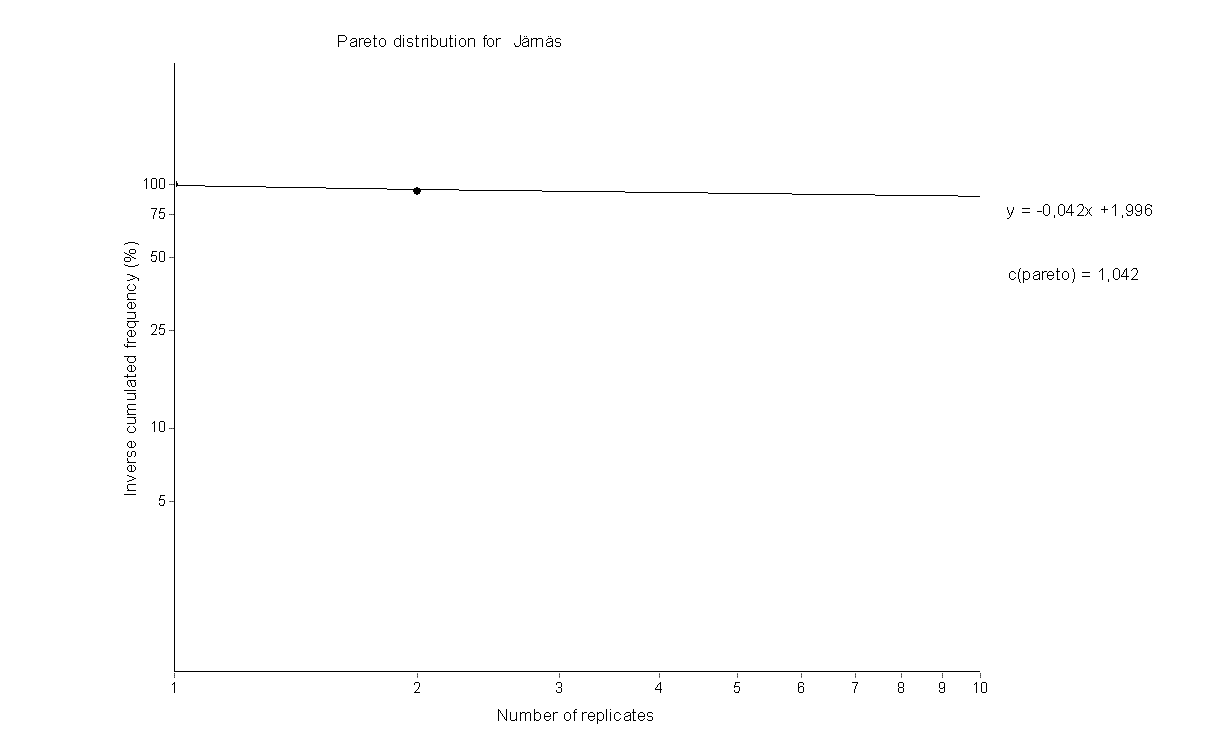
**

**Fig. S3 C**

**Tables**

**Table S1. Pairwise *F_ST_*-values of *F. radicans.***

| SITE | Fin A | Fin B | Fin C | Fin D | Swe E | Swe F | Swe G | Est H | Est I | Swe J | Swe K | Swe L | Swe M | Swe N | Swe O |
| --- | --- | --- | --- | --- | --- | --- | --- | --- | --- | --- | --- | --- | --- | --- | --- |
| Fin B | 0.01 |  |  |  |  |  |  |  |  |  |  |  |  |  |  |
| Fin C | 0.05 | 0.04 |  |  |  |  |  |  |  |  |  |  |  |  |  |
| Fin D | 0.16 | 0.12 | 0.00 |  |  |  |  |  |  |  |  |  |  |  |  |
| Swe E | 0.12 | 0.08 | 0.00 | 0.03 |  |  |  |  |  |  |  |  |  |  |  |
| Swe F | 0.11 | 0.09 | 0.05 | 0.13 | 0.02 |  |  |  |  |  |  |  |  |  |  |
| Swe G | 0.13 | 0.11 | 0.02 | 0.09 | 0.02 | 0.03 |  |  |  |  |  |  |  |  |  |
| Est H | 0.22 | 0.23 | 0.22 | 0.36 | 0.25 | 0.32 | 0.23 |  |  |  |  |  |  |  |  |
| Est I | 0.22 | 0.25 | 0.21 | 0.38 | 0.30 | 0.28 | 0.26 | 0.01 |  |  |  |  |  |  |  |
| Swe J | 0.08 | 0.05 | 0.02 | 0.13 | 0.02 | 0.00 | 0.02 | 0.22 | 0.24 |  |  |  |  |  |  |
| Swe K | 0.14 | 0.10 | 0.00 | 0.00 | 0.00 | 0.07 | 0.05 | 0.32 | 0.33 | 0.07 |  |  |  |  |  |
| Swe L | 0.11 | 0.08 | 0.00 | 0.01 | 0.01 | 0.06 | 0.06 | 0.31 | 0.31 | 0.05 | 0.00 |  |  |  |  |
| Swe M | 0.06 | 0.04 | 0.02 | 0.07 | 0.05 | 0.08 | 0.09 | 0.28 | 0.28 | 0.03 | 0.06 | 0.03 |  |  |  |
| Swe N | 0.13 | 0.08 | 0.02 | 0.00 | 0.04 | 0.12 | 0.09 | 0.32 | 0.33 | 0.12 | 0.02 | 0.04 | 0.06 |  |  |
| Swe O | 0.13 | 0.08 | 0.02 | 0.03 | 0.04 | 0.09 | 0.08 | 0.32 | 0.35 | 0.08 | 0.03 | 0.05 | 0.06 | 0.00 |  |
| Swe P | 0.12 | 0.07 | 0.04 | 0.06 | 0.01 | 0.04 | 0.04 | 0.28 | 0.31 | 0.04 | 0.02 | 0.06 | 0.07 | 0.01 | 0.00 |

Pairwise *F_ST_*-values of of population differentiation of 16 *F. radicans* sites at the genet level in the Baltic Sea. All *P-*values that remained significant after Bonferroni correction are shown in grey.
